# Mitochondrial dysfunction in acute and post-acute phases of COVID-19 and risk of non-communicable diseases

**DOI:** 10.1038/s44324-024-00038-x

**Published:** 2024-12-04

**Authors:** Helena Borland Madsen, Jon Ambæk Durhuus, Ove Andersen, Per thor Straten, Anne Rahbech, Claus Desler

**Affiliations:** 1 Department of Biomedical Sciences, University of Copenhagen, Copenhagen, Denmark; 2 Department of Clinical Research, Copenhagen University Hospital—Amager and Hvidovre, Hvidovre, 2650 Copenhagen, Denmark; 3 National Center for Cancer Immune Therapy, Department of Oncology, University Hospital Herlev, Copenhagen, Denmark

**Keywords:** Diseases, Mitochondria

## Abstract

The COVID-19 pandemic, caused by SARS-CoV-2, has resulted in widespread morbidity and mortality, with a significant portion of the affected population experiencing long-term health complications. This review explores the mechanisms of mitochondrial dysfunction in both the acute and post-acute phases of COVID-19, highlighting its impact on various organs and its potential role in the development of non-communicable diseases (NCDs). We discuss how SARS-CoV-2 directly affects mitochondrial function and the role of the virus-induced immune response in exacerbating mitochondrial impairment. This review highlights the critical role of mitochondria in COVID-19 pathogenesis and the importance of addressing mitochondrial health to mitigate acute and chronic effects of the disease.

## Introduction

During the acute phase of corona virus disease 2019 (COVID-19), the primary symptoms of the infection are respiratory illness and gastrointestinal symptoms^1,2^. More severe complications, include multi-organ failure^3^ that can manifest as acute respiratory distress syndrome (ARDS), heart problems, kidney damage, liver dysfunction, and neurological issues, potentially resulting in significant morbidity and mortality^4^. In some people, after recovery, the effects of the first waves of COVID-19 persist beyond the acute phase and increase the risk of chronic multiorgan symptoms and disease^5,6^. Up to 70% of people affected by COVID-19 show reduced organ function even four months or more after COVID-19 diagnosis^5,6^. Such a functional decline is associated with an increased risk of the development of non-communicable diseases (NCDs)^7^.

SARS-CoV-2, the virus that causes COVID-19, is an enveloped single stranded positive-sense RNA beta-corona virus that encodes four structural and 25 non-structural proteins^8^. SARS-CoV-2 host cell primary entry is facilitated by the viral spike protein, where transmembrane serine proteases (TMPRSS) enable attachment to the host cell surface angiotensin-converting enzyme 2 (ACE2) receptor. While the primary sites of infection include the nasal cavity and lung epithelium, viral particles from SARS-CoV-2 infection have been observed in multiple tissues. ACE2 distribution to provide direct viral entry to, for example, the heart, kidney and gastrointestinal tract combined with hyperinflammation may aid SARS-CoV-2 to become a multiorgan pathogenesis (as reviewed elsewhere^9^).

Even though the etiology of COVID-19 is complex and there are multiple factors involved, decreased organ function has been associated with mitochondrial dysfunction for both the acute phase and post-acute phase of COVID-19^10–20^. We define the post-acute phase as the period four months or more following the acute-phase of COVID-19. This definition does not distinguish whether the patient is symptomatic or asymptomatic. Here we will review the known mechanisms leading to impaired mitochondrial function in the acute phase as well as in the post-acute phase of COVID-19 and discuss the relevance of decreased mitochondrial function for the increased risk of diseases including NCDs.

### Immune response to infection

The innate and adaptive immune systems play crucial roles in combating SARS-CoV-2 infection. while both the virus and the immune response impact mitochondrial health. Understanding the primary immune responses to SARS-CoV-2 infection is therefore essential for elucidating the mechanisms underlying COVID-19-induced mitochondrial dysfunction.

#### The Innate immune response to SARS-CoV-2

Inflammation can be triggered by infection or tissue injury when pattern recognition receptors (PRRs) respond to damage-associated molecular patterns (DAMP) or pathogen-associated molecular pattern (PAMP). One type of PRRs are Toll-like receptors (TLRs), which are expressed on the cell surface of various immune cells and play a key role in the first part of an immune response. Mediated by the innate immune system, TLR activation leads to an increase in pro-inflammatory proteins, adhesion molecules, and matrix metalloproteinases. TLR2 and TLR4 recognize SARS-CoV-2 envelope and spike protein respectively, whereas TLR3 and TLR7 recognize the viral RNA, thus activating a pro-inflammatory immune response^21^.

**Fig. 1:**
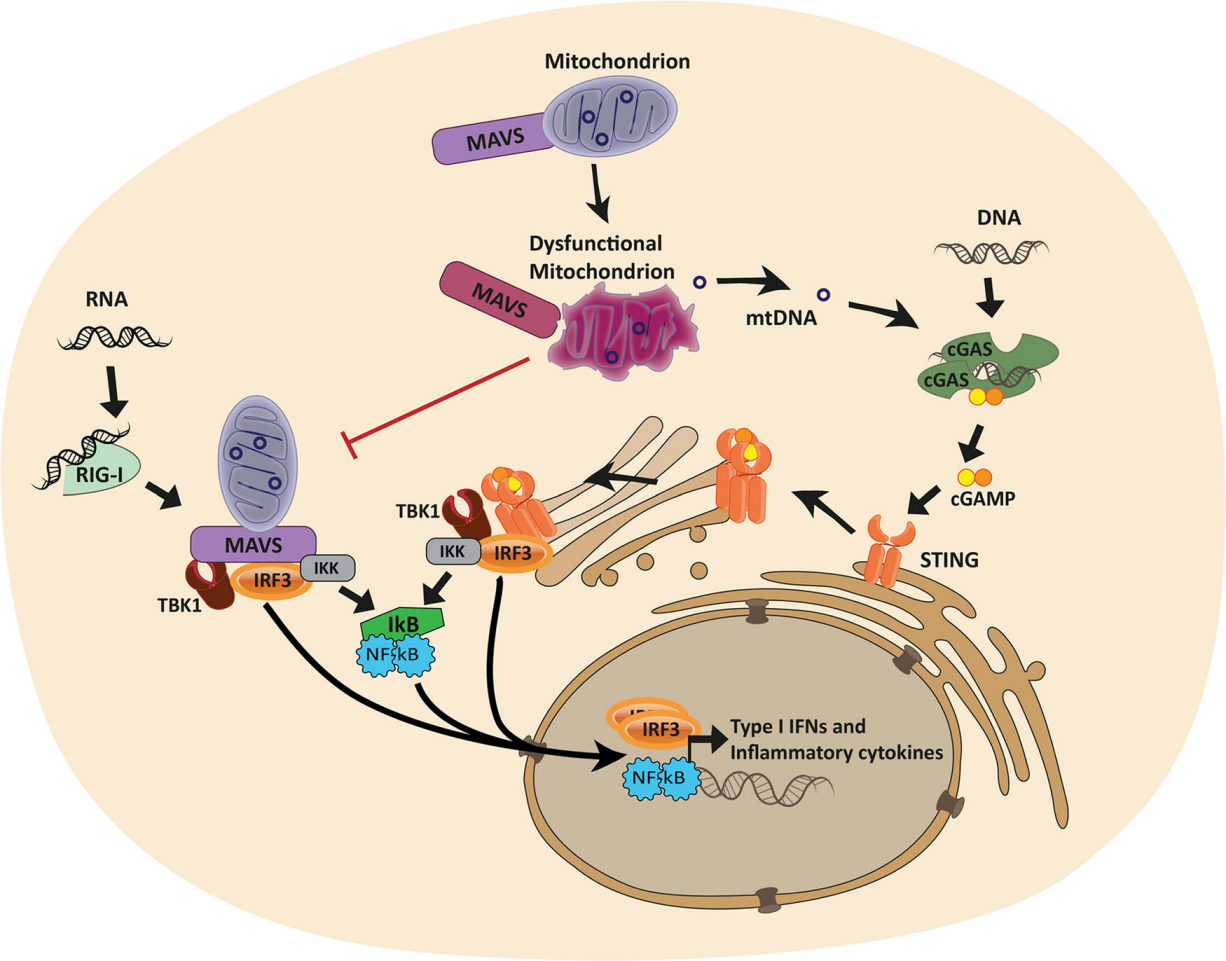
The role of mitochondria in innate immune activation of DNA- and RNA-sensing pathways. Cytosolic RNA can activate retinoic acid-inducible gene I (RIG-I), enabling Mitochondrial antiviral signaling protein (MAVS) to bind and oligomerize, thereby allowing TANK-binding kinase 1 (TBK1) to bind and phosphorylate MAVS. Downstream Interferon regulatory factor 3 (IRF3) is activated and translocates to the nucleus to induce the transcription of type 1 IFN genes. Likewise, cytosolic DNA can activate stimulator of interferon response cGAMP interactor 1 (STING) via the second messenger 2'3’-cyclic GMP-AMP (cGAMP) produced by cyclic GMP–AMP synthase (cGAS). STING then translocates from the endoplasmatic reticulum to the golgi to bind TBK1, and subsequently activates IRF3. Alternatively, both pathways can lead to activation of Nuclear Factor Kappa B (NF-Ƙb) to orchestrate a more pro-inflammatory response. Mitochondria play a dual role in these pathways, as they host the MAVS protein, which activation is directly dependent on mitochondrial health, and mitochondrial DNA (mtDNA) can be released from compromised mitochondria to activate DNA-sensing pattern recognition receptors such as cGAS.

Mitochondria are now being recognized as central players in propagating innate immune responses via PRRs including DNA- and RNA-sensors^22^. The intracellular DNA-sensor, cyclic GMP–AMP synthase (cGAS), scavenges the cytosol for both foreign- and self- double-stranded DNA, including mitochondrial DNA (mtDNA). It has been shown that mitochondrial and bacterial nucleoid proteins can stimulate DNA-sensing by cGAS, whereas histone-bound DNA, such as nucleic DNA, is more protected from cGAS activation^23,24^. Upon detection, cGAS generates 2'3’-cyclic GMP-AMP (cGAMP), a second messenger, which activates stimulator of interferon response cGAMP interactor 1 (STING) (Fig. 1). STING is located in the ER but translocates to the Golgi/ERGIC upon cGAMP stimulation to bind TANK-binding kinase 1 (TBK1). The STING/TBK1 dimer oligomerizes, enabling TBK1 to trans-autophosphorylate^25^. Activated TBK1 then phosphorylates STING, enabling binding, activation and subsequent nuclear translocation of the Interferon regulatory factor 3 (IRF), which is a type 1 IFN gene transcription factor. Post STING activation, STING is degraded via autophagy, a process known to aid cytosolic clearance of dsDNA^26^.

Like the DNA sensing pathway, cGAS-STING, cells contain a similar transduction pathway for the detection of RNA via retinoic acid-inducible gene I (RIG-I)^27,28^ among other RNA sensors. While RIG-I is activated via double-stranded RNA, and SARS-CoV-2 is a single-strand virus, double-stranded RNA has been identified in double-membrane vesicles of infected cells, considered to be viral replication intermediates^29^. A pore complex has been identified^30^ which may allow these viral double-stranded RNAs to enter the cytosol for PRR activation directly by the virus. Furthermore, viral double-stranded RNA has also been identified inside mitochondria^31^, from where they can be released, together with mtDNA, upon mitochondrial dysfunction and membrane compromisation. Importantly, SARS-CoV-2 is sensed by RIG-I in both lung and intestinal epithelial cells, driving inflammation during infection^32,33^. While double-stranded mitochondrial RNA (mtRNA) can activate several PRRs^34,35^, mitochondria play a dual role in the RIG-I pathway, as they can provide mtRNA ligands for RIG-I activation, but they also host the downstream mitochondrial antiviral-signaling protein (MAVS) in their outer membrane^36^. Upon binding of dsRNA, RIG-I, undergoes conformational changes, allowing MAVS binding. Upon interaction, MAVS oligomerizes, enabling TBK1 to bind and phosphorylate MAVS. Like STING, phosphorylated MAVS recruits IRF3 for activation by phosphorylation and subsequent translocation to the nucleus, to induce transcription of type 1 IFN genes. Alternatively, both cGAS-STING and RIG-I/MAVS pathways can activate Nuclear Factor Kappa B (NF-Ƙb) resulting in release of pro-inflammatory cytokines^36,37^.

MAVS’ localization to the mitochondrial membrane demonstrates another direct link between immune signaling and mitochondrial health, as MAVS signaling is inhibited by both excessive mitochondrial fission^38^ and fusion^39^. Furthermore, the mitochondrial membrane potential is required for an efficient type 1 IFN response^40^.

#### The Adaptive immune response to SARS-CoV-2

Following viral infection, all three main types of lymphocytes in the adaptive immune system will be activated: CD4+ T cells, CD8+ T cells, and antibody-producing B cells. Effective clearance of the virus is highly dependent on both CD4+ and CD8+ T cells and reduced total numbers of T cells correlate with more severe cases of COVID-19 severity^41^. The main role of CD4+ T cells is to support the activation and differentiation of CD8+ T cells and B cells. CD8+ T cells produce cytotoxic molecules, such as interferon gamma (IFNγ), CD107, perforin, and granzyme B, which can directly eliminate infected cells. As an essential component of the humoral part of the adaptive immune response, activated B cells facilitate production of neutralizing antibodies and initiate antibody-dependent cellular cytotoxicity^42^. The latter involves natural killer (NK) cells and macrophages as key effector cells, thus linking back to the innate immune system.

#### Cytokine response

A fundamental part of an immune response against viral infection is the release of numerous soluble immune-related molecules, such as cytokines and chemokines. As a part of the innate immune response to COVID-19 the NLRP3 inflammasome is generated, which stimulates the release of interleukin 1β (IL-1β), IL-18, and gasdermin D (GSDMD), ultimately leading to pyroptosis of the infected cell^43^. Furthermore, type 1 IFNs and proinflammatory cytokines are released upon viral nucleic acid sensing as described above. Other inflammatory cytokines will be released as a response to the acute phase of COVID-19 (see Table 1)^44–46^. Some of them appear to have an essential role in disease development, such as IL-6. Elevated expression of IL-6 has been correlated to a poor prognosis for COVID-19 patients in several studies^47,48^. IL-6 is involved in class switch recombination, but also in T cell exhaustion. In line with this, IL-10, which is involved in the same processes as IL-6, is also reported to be increased in the plasma of patients with acute COVID-19^49^. Large cohort studies exploring plasma cytokines further revealed increased levels of the chemokine receptor C-X-C motif chemokine ligand 10 (CXCL10) (IP-10), IL-2R, tumor necrosis factor α (TNFα), and IL-8 in severe cases of COVID-19^41,45^.

**Table 1:** Cytokines, chemokines and others known from literature to be elevated in the acute and post-acute phase

Acute phase	Cytokines: IL-2R, IL-1β, IL-6, IL-10, IL-12, IL-18, TNFα and IFNγ
	Chemokines: CCL2, CCL3, CCL7, CCL8, CXCL-10
	Others: GM-CSF, MCP-1
Post-acute phase	IL-6, TNFα, CXCL-10, IL-1β

#### Hyperactivation of the immune system

Activation of the pro-inflammatory immune response upon viral entry needs to be tightly regulated. If not, it might lead to a state of hyperinflammation and cytokine storm. The phenomenon of a cytokine storm has been shown to be a key aspect in severe cases of COVID-19^46^. When excessive cytokines are secreted, overstimulation of the T cells will occur, leading to exhaustion and impaired function. This will in turn increase the risk of new infections. Overall, hyperactivation of the immune system can contribute to a state of severe COVID-19 including lung damage, ARDS, organ failure, and even death^50^. Immune checkpoints have a central role in controlling the immune response. However, the virus might circumvent these immune checkpoints, resulting in increased activation and exhaustion of the cells. Hence, treatment using immune checkpoint inhibitors (ICI) is being investigated^51^. It has been proposed that a combination of a reduced innate immune response and excessive cytokine production are the driving force of COVID-19^52^.

#### Long-term dysregulation of the immune system in COVID-19

The hyperactivated immune system might not only lead to severe acute COVID-19 symptoms, but it could also be an underlying cause of post-acute sequelae of COVID-19 (PASC) also referred to as”long COVID”. A large study based on samples from patients with long COVID revealed systemic inflammation and immune dysregulation^53^. Several other studies have found elevated levels of various plasma inflammatory markers in long COVID (see Table 1). One study observed an elevated level of TNFα and CXCL-10 in an early stage in patients who developed long COVID. In addition, IL-6 was elevated in the later recovery^54^. Another study aimed to identify the cytokine profile of long COVID patients and revealed elevated levels of IL-1β, IL-6, and TNFα plasma levels. In addition, no association between autoantibodies and long COVID symptoms was found. They further suggested overactivated monocytes to be the likely cause of the increased cytokine production, due to the generation of positive feedback loops^55^. However, the underlying mechanism of systemic inflammation in long COVID patients is unclear. Another hypothesis is that insufficient viral clearance creates a viral reservoir, resulting in chronic activation of immune cells. It leads to a change in the distribution of cell phenotypes, with a higher fraction of terminally differentiated cells and less naïve T and B cells^56^. Thus, management of long COVID complications seems to be a balancing act; the risk of preventing tissue damage and disease progression at the cost of continued or prolonged infection.

### Observations of mitochondrial dysfunction in the acute and post-acute phase of COVID-19

In the acute phase of COVID-19, mitochondrial dysfunction has been observed across various tissues. In the lungs, COVID-19 patients exhibit impaired mitochondrial coding and regulation of gene expression, affecting mitochondrial function and related metabolic pathways^10^. Similarly, autopsy studies of COVID-19 patients reveal a suppression of transcription for mitochondrial genes encoded by both nuclear and mtDNA, along with an induction of glycolysis in heart^20^. In autopsies of kidneys, proteomic analysis reveals a downregulation of oxidative phosphorylation^11^. Additionally, alterations in mitochondrial ultrastructure have been consistently observed in autopsies of the lungs, heart, liver, and kidneys of patients who died from COVID-19, indicating widespread mitochondrial impairment^12^.

The impaired mitochondrial function of the acute phase of COVID-19, has been observed to persist in the post-acute phase of the disease. Here, oxidative phosphorylation in the heart, kidney, liver, and lymph nodes remained impaired in autopsied tissue even after the viral load is eliminated^20^. For the post-acute phase of COVID-19, the majority of studies have focused on patients suffering from long COVID. Long COVID covers an extensive spectrum of symptoms including fatigue and shortness of breath, and many patients experience exercise intolerance, as reviewed in ref. ^13^. Notably, these symptoms closely resemble those observed in mitochondrial diseases. Likely many different effectors are responsible for these symptoms, nevertheless, several studies have investigated and found mitochondrial dysfunction to be a part of the etiology. Peripheral blood mononuclear cells (PBMCs) isolated from long COVID patients demonstrated significantly impaired properties of oxidative phosphorylation, where especially the basal respiration and reserve capacity were reduced to half of healthy controls^57^. Significant impairment of mitochondrial functional and morphological structure has been demonstrated in skeletal muscle biopsies from long COVID patients when compared to matched controls^15,16^. Furthermore, the development of post-COVID-19 pulmonary complications has been associated with imbalance of serum levels of the mitochondrial proteins PINK1, DNM1L, and MFN2 indicating an involvement of mitochondrial fission, fusion and mitophagy^58^.

Few studies have focused on the post-acute phase of COVID-19. The literature suggests that there is a difference in mitochondrial function between patients suffering from long COVID and asymptomatic individuals having recovered from COVID-19^17,18^. Here, effects on mitochondria are reported to be more severe on patients with long COVID. Nevertheless, a comparative study of patients suffering from long COVID, and patients fully recovered from COVID-19, demonstrated a metabolic phenotype with dysfunctional mitochondria-dependent lipid catabolism for both groups, when compared to persons with no history of COVID-19^17^. Here the post-acute phase was defined as at least 28 days after testing positive for SARS-CoV-2. A separate study used mitochondrial membrane potential as a marker for mitochondrial fitness. Here PBMCs isolated from patients in the acute phase of COVID-19, PBMCs isolated approximately a month, and a year after the acute phase, demonstrated a significantly lower mitochondrial membrane potential when compared to PBMCs isolated from uninfected controls^19^.

### Direct effect of SARS-CoV-2 on mitochondria

The deleterious effects of COVID-19 on mitochondrial function can be elicited directly in the acute phase by the virus itself, or indirectly through the virus-induced immune response.

Mitochondria play a specific role in the pathogenesis of some infectious agents, like Mycobacterium tuberculosis^59^, type 1 herpes simplex virus (HSV-1)^60^, dengue virus^61^ and also SARS-CoV-2^62^. In the case of SARS-CoV-2, it was shown that mitochondria appear swollen and damaged in skin biopsies from patients, and that depletion of mtDNA in endothelial cells significantly reduced the type I IFN response^62^. In SARS-Cov-2 infected cells, viral RNA was found to be associated with mitochondria using fluorescence and electron microscopy^31^. Several reports have suggested the colocalization of mitochondrial-SARS-CoV-2 RNA in infected human tissue^63^. SARS-CoV-2 proteins have been documented to interact with several host proteins, many of which are mitochondrial. For example, the structural protein membrane (M) has been demonstrated to bind to several mitochondrial proteins including leucyl-tRNA synthase, asparaginyl-tRNA synthase and complex I subunits^20^, which may cause apoptosis via mitochondrial release of the DAMP, cytochrome c^64^. The non-structural proteins NSP6 binds to complex V subunits, open reading frame (ORF)10 binds to translocase of inner mitochondrial membrane (TIMM)8 and ORF9b to translocase of outer mitochondrial membrane (TOMM)70^65–68^. The presence of viral particles near the mitochondrial matrix is hypothesized to contribute to mitochondrial dysfunction, increased ROS, and compromised membrane integrity, potentially leading to cell death^69,70^. The release of degraded mitochondria is associated with poor outcome in COVID-19^71^. ORF3b accumulates in nucleus and mitochondria of SARS-CoV-2 infected cells, blocks cell growth and inhibits IFN^72^. Although these findings support the hypothesis of SARS-CoV-2’s involvement in mitochondrial dysregulation, ongoing research is essential to further understand the implications of these mitochondrial-virus interactions.

SARS-CoV-2 can use mitochondria to evade host defense mechanisms. Several SARS-CoV-2 proteins have been identified to target the RIG-I/MAVS innate immune pathway directly^73^, such as the ORF-9b which triggers the degradation of MAVS^39^ or NSP5 which truncates RIG-I thus inactivating MAVS^74^ to evade host innate immunity. Another example is SARS-CoV-2 ORF10, which both target degradation of MAVS via mitophagy stimulation^75^, and interacts with STING to prevent translocation and inhibit STING-TBK1 binding and downstream signaling^76^. Thus, SARS-CoV-2 targets both mitochondria directly, and their ability to regulate the immune response.

### Infections indirectly alter mitochondrial function via inflammatory cytokines

While inflammation is induced to clear viral load and protect host cells, this process is not without consequences for the infected tissues. Among the inflammatory mediators induced by COVID-19 (see Table 1), two of the major pro-inflammatory cytokines, IL-1β and TNFα are each capable of causing mtDNA damage and dysfunction, decreasing ATP levels in cells^77^. Early literature has shown that TNFα treatment can cause a rapid inhibition of the electron transport chain within one hour of treatment to mouse fibrosarcoma cells^78^ and induces mitochondrial ROS-mediated apoptosis^79,80^. Both TNFα and IL-1α decrease pyruvate dehydrogenase activity in cardiomyocytes, with parallel decreased oxygen consumption of the respiratory chain complex I and II^81^. IL-6, which is also expressed in both the acute and post-acute phase of COVID-19, induces expression of the mitochondrial fission proteins Dynamin-1-like protein and mitochondrial fission 1 protein in skeletal muscles cells and mice^82^. IL-6 also increases ROS and oxygen consumption in skeletal muscle cells, which is followed by decreased mitochondrial respiration upon sustained exposure^83^, underlining the dynamic role of mitochondria in stress responses. While some of these mitochondrial alterations are part of the complex regulation to orchestrate a proper immune response, their combination, or prolonged effect can obstruct the mitochondria and exacerbate inflammation.

### Mitochondrial dysfunction linked to innate immune activation and disease

Mitochondria are essential in all human cell types except the red blood cells. STING is ubiquitously expressed in several tissues including the heart, kidney and lung tissues^84^ and thus, dysfunctional mitochondria and subsequent activation of the innate immune system can injury a range of tissues and result in a plethora of diseases.

cGAS-STING pathway activation has been associated with acute kidney injury in humans and can be ameliorated by STING knock-out/inhibition^85–88^ or by cGAS knock-out^89^ in mouse models of acute kidney injury. mtDNA leakage into the cytosol has been observed in several mouse models of kidney injury^86,87,89^ where depletion of mtDNA suppressed inflammation^86^, suggesting that mitochondrial dysfunction and subsequent activation of the cGAS-STING pathway is critical in kidney injury. In correlation, mtDNA released into urine can be used as a marker for progressing acute kidney injury^90^ associated with renal dysfunction as its level positively correlates with serum creatinine level and negatively correlates with glomerular filtration rate^91^. Finally, STING silencing in a lipopolysaccharide (LPS)-induced mouse model of acute kidney injury has also shown to benefit mitochondria, by increasing the mitochondrial membrane potential and ATP level, while decreasing mitochondrial ROS^88^. LPS is a PAMP that is recognized by TLRs to initiate host immune responses^21^. In a mouse model of chronic kidney disease, vascular smooth muscle cells were shown to sense oxidative stress-induced mitochondrial damage via the cGAS-STING pathway, and the following type 1 IFN response could be attenuated by knocking out STING or cGAS^92^.

Cardiovascular diseases are the leading cause of death worldwide, and incidents have increased by almost 20% over the past decade as estimated by the American heart association^93^. Cardiovascular diseases are multifaceted but share common elements such as inflammation and fibrosis. In several cardiovascular disease models, for example of myocardial infarction^94^, aortic aneurysm and dissection^95^, heart failure^14,96^ and diabetic cardiomyopathy^97,98^ leakage of DNA into the cytosol to activate cGAS-STING has been observed. Several of these studies directly identify mtDNA as the cytoplasmic inducer of inflammation^14,97,98^, and/or identifies mitochondrial ROS as a potential trigger of DNA damage and leakage^95,98^ placing mitochondrial dysfunction as a central player in cardiovascular disease.

Incidents of immune response-mediated acute lung injury or the more severe form, respiratory distress syndrome, are associated with severe SARS-CoV-2 infection^99^.

Circulating mtDNA has been shown to enhance acute lung injury via cGAS-STING signaling in mice, and interestingly, STING activation was found to disturb autophagic clearance^100^, which is otherwise known to be stimulated downstream of STING activation^26^. In lung fibroblasts from pulmonary fibrosis patients the level of cytosolic mtDNA was increased compared to control cells, and mediated senescence in a cGAS-dependent manner^101^.

Interestingly, levels of plasma mtDNA is associated with the incidence and severity of acute lung injury and respiratory distress syndrome in patients suffering from sepsis and trauma^100,102^.

In summary, mitochondrial dysfunction followed by mtDNA release to trigger inflammation has been implicated in a wide range of diseases, and thus stands as an imperative therapeutic pathway for future research.

## Discussion

Both the acute and post-acute phases of COVID-19 are associated with decreased organ function, implying an increased risk of NCDs^10–20^. In this review, we have explored the potential involvement of mitochondrial dysfunction in the etiology of these phases. COVID-19 is a complex and multifaceted disease, and its impact on organ functionality results from a combination of factors that cannot be attributed to mitochondrial dysfunction alone. Nonetheless, the role of mitochondria in this context deserves attention, as it adds to our current understanding of the acute and post-acute phases of COVID-19. Moreover, it paves the way for the future identification of potential biomarkers and therapeutic targets. Here, we have reviewed the observations of mitochondrial dysfunction in the pathology of COVID-19. During the acute phase, mitochondrial dysfunction has been demonstrated to be directly correlated with viral entry into the mitochondria of the host cells^31,67^. Both in the acute and post-acute phases, the impairment of mitochondrial function is linked to the immune response of the infection^20^. Observations have associated cGas-Sting activation via mtDNA release to COVID-19^62^, a pathway known to be activated in various diseases, including kidney^86,87,89^, heart^14,97,98^, and lung^100,101^ diseases. This constitutes a credible correlation between the acute, and the post-acute phase of COVID-19 and decreased organ function. Furthermore, inflammatory signaling via the RIG-I pathway is dependent on mitochondrial health^38–40^ and plays a key role in the inflammatory response to SARS-CoV-2 infection^32,33^. The importance of these innate immune pathways is highlighted by the multitude of evasion strategies SARS-CoV-2 has evolved to evade their activation^39,75,76^. Blocking the acute innate immune response during infection, allowing continued viral replication and ongoing attraction of immune cells to damage the microenvironment, may create the necessary conditions for sustained and excessive inflammation in the post-acute phase, via release of DAMPs, including mtDNA and mtRNA, from the damaged tissues^62,103,104^.

Likely other mitochondrial pathways are also involved, highlighting the importance of continued research in this area. In this review, we discern between the post-acute phase and the condition of long COVID. As reviewed, most studies have focused on patients suffering from long COVID. In the few studies comparing patients with long COVID, to asymptomatic individuals who survived COVID-19, significant differences in the severity of mitochondrial impact have been found^17–19^. Still, same studies also report more impaired mitochondrial function in the asymptomatic group compared to healthy non-infected controls. Based on this, we hypothesize that the mitochondrial dysfunction is a complicit factor for the increased risk of impaired organ function in the post-acute phase of COVID-19, regardless of whether the individual suffers long COVID. We recognize that much work is needed to demonstrate the true impact of mitochondrial dysfunction in the post-acute phase of COVID-19 and how this knowledge can help reduce the risk of developing NCDs. Future research should aim to further elucidate the mechanisms of mitochondrial involvement and identify potential therapeutic strategies to mitigate the long-term health impacts of COVID-19. Additionally, it is crucial to investigate the effects of drugs administered during the acute and post-acute phases on mitochondrial function, as these treatments may contribute to or exacerbate mitochondrial dysfunction. Understanding these interactions will be key to developing comprehensive therapeutic approaches that address both the acute management of COVID-19 and the prevention of long-term complications.

Finally, while this review focuses on COVID-19, it is important to consider that other severe viral infections may follow a similar path regarding mitochondrial impairment and the risk of NCDs. Understanding the role of mitochondrial dysfunction in the context of COVID-19 can thus provide valuable insights applicable to other viral infections. Viruses such as influenza^105^, RSV^106^ and others have been known to cause significant organ damage and prolonged health issues. Therefore, the study of mitochondrial impairment in viral infections holds broader relevance, offering the potential to inform therapeutic approaches and mitigate the long-term health impacts across various viral diseases. This underscores the necessity for continued research into mitochondrial health and its implications for viral pathologies, ensuring we are better prepared for future outbreaks.

## Data Availability

No datasets were generated or analyzed during the current study.
